# Enhancing Safety and Efficiency in Firefighting Operations via Deep Learning and Temperature Forecasting Modeling in Autonomous Unit †

**DOI:** 10.3390/s23104628

**Published:** 2023-05-10

**Authors:** Adenrele A. Ishola, Damian Valles

**Affiliations:** Ingram School of Engineering, Texas State University, San Marcos, TX 78666, USA

**Keywords:** deep learning, autoencoder, ANN, ARIMA, random forest regression, firefighting, fire

## Abstract

Firefighters face numerous challenges when entering burning structures to rescue trapped victims, assess the conditions of a residential structure, and extinguish the fire as quickly as possible. These challenges include extreme temperatures, smoke, toxic gases, explosions, and falling objects, which can hinder their efficiency and pose risks to their safety. Accurate information and data about the burning site can help firefighters make informed decisions about their duties and determine when it is safe to enter and evacuate, reducing the likelihood of casualties. This research presents unsupervised deep learning (DL) to classify the danger levels at a burning site and an autoregressive integrated moving average (ARIMA) prediction model to forecast temperature changes using the extrapolation of a random forest regressor. The DL classifier algorithms provide the chief firefighter with an awareness of the danger levels in the burning compartment. The prediction models forecast the rise in temperature from a height ranging from 0.6 m to 2.6 m and the changes in temperature over time at an altitude of 2.6 m. Predicting the temperature at this altitude is critical as the temperature increases faster with height, and elevated temperatures can weaken the building’s structural material. We also investigated a new classification method using an unsupervised DL autoencoder artificial neural network (AE-ANN). The prediction data analytical approach included using the autoregressive integrated moving average (ARIMA) with random forest regression implementation. The proposed AE-ANN model, with an accuracy score of 0.869, did not perform as well compared to previous work, with an accuracy of 0.989, at achieving high accuracy scores for the classification task using the same dataset. However, the random forest regressor and our ARIMA models are analyzed and evaluated in this work, while other research has not utilized this dataset, even though it is open-sourced. However, the ARIMA model demonstrated remarkable predictions of the trends of temperature changes in a burning site. The proposed research aims to classify fire sites into dangerous levels and predict temperature progression using deep learning and predictive modeling techniques. This research’s main contribution is using a random forest regressor and autoregressive integrated moving average models to predict temperature trends in burning sites. This research demonstrates the potential of using deep learning and predictive modeling to enhance firefighter safety and decision-making processes.

## 1. Introduction

The level of safety risk differs with the occupation, and firefighting is a high-risk task. Firefighters are more prone to be injured and possibly killed while doing their jobs compared to other workers. They put their lives in danger to rescue victims (humans and pets) from burning structures and dangerous situations. The National Fire Protection Association (NFPA) reported that out of an estimated 58,250 firefighter injuries in the line of duty in 2018, 22,975 injuries (39%) happened at the fire site [1]. These first responders were exposed to highly dynamic environments, including elevated temperatures and convective and radiant thermal flux, significantly affecting the firefighters’ protective equipment (PPE). There are numerous ways that a firefighter can be injured on the job at the fire site: strains and sprains caused 38% of the above injuries; 13% percent were caused by gas inhalation; 11% were caused by wounds, cuts, bleeding, and bruises; and 10% percent were caused by thermal stress reported by the NFPA [1]. Research showed that one of the causative factors in individual firefighters’ death and injuries is thermally degraded and melted self-contained breathing apparatus (SCBA) facepieces [2].

Modern residential buildings and businesses contain tons of synthetics, plastics, and chemical materials that increase the rate of explosions in a burning building. These explosions also coat firefighters with toxic soot. According to the NFPA, a rapid-fire blast is the second highest cause of firefighter fatalities, with about six deaths or 13% of the fatality rate annually, and is only behind overexertion, which causes 26 deaths annually, or 54% of the fatality rate [3]. Explosions pose a significant danger to firefighters because they have little or no knowledge of when they occur. With all these uncertainties while carrying out their task, it is crucial to provide the firefighters with informed data about the fire scene to assist in making vital decisions about when to evacuate the burning compartment.

Current ways to improve firefighters’ safety involve providing data on the firefighters’ situations to make informed decisions. The data could be collected and made available by furnishing buildings with sensors and methods of communication between facilities and introducing the smart city concept to firefighting. Smart firefighting is regularly improved by integrating recent artificial intelligence (AI) techniques [2,4,5]. The research conducted in [6] designed an autonomous embedded system vehicle (AESV) installed with different sensors, including light detection and ranging (LiDAR) and a global positioning system (GPS), to navigate burning sites autonomously; capture environmental, imaging, and audio data; and transmit it to a base station in a fire bus outside the burning area for analysis. The vehicle measures approximately from four to six inches in height, inhibiting its capability to capture temperature at the ceiling. The unit consists of a Teensy 3.6 board by which the sensor peripherals process the collected data and communicate with the NVIDIA Jetson Nano over a USB connection. The Jetson runs the Robotic Operating System 2 (ROS2) for data telemetry and navigation.

Extreme temperatures, low visibility, falling objects, toxic air pollutants such as Carbon Monoxide (CO), and the uncertainty in the fire ground make rescuing victims difficult [7]. These factors increase firefighters’ risk of injury, safety, and long-term health issues. Smoke inhalation for a short time can cause acute effects. Research shows that people exposed to heavy smoke experience temporary changes in lung function, making breathing more difficult. Smoke contains two primary agents, CO and fine particles, or PM2.5, that cause health effects. CO inhalation reduces the body’s oxygen supply, leading to headaches and exacerbating a heart condition known as angina. Firefighters’ protective equipment includes SCBA, which offers minimal protection because it melts at 300 °C [2].

To tackle the abovementioned challenges and increase firefighters’ safety on the fire site, it is necessary to develop a solution that enhances the firefighters’ situational awareness by exploiting the environmental data actively captured by the AESV on the scene. This solution uses DL models to classify the data into dangerous levels and predicts temperature at the height of 2.6 m, where sensor data cannot be collected. This solution can accurately inform the decision-making process of firefighters with real-time scene information by processing and analyzing essential data. Utilizing the output of this framework, firefighters can effectively decide when it is safe to enter and evacuate the fire ground with minimal health and safety risk.

**Figure 1:**
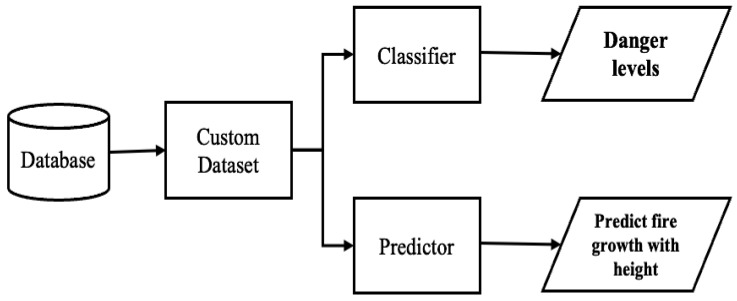
Solution for predicting firefighters’ safety.

Figure 1 depicts the overview scope of the presented work. This work aims to improve firefighters’ safety during victims’ evacuation from burning sites. This solution develops an algorithm to generate a custom dataset from a database of environmental data captured in a burning area by considering the AESV unit’s possible movements. The solution also uses deep learning (DL) models to classify the burning site into three danger levels and prediction models on environmental data to predict the temperature at heights closer to the ceiling and the effect of time on temperature. The classifiers use the impact and influence of ecological factors on the firefighters’ PPE to classify the danger level of the fire site. At the same time, the predictor forecasts the fire growth at heights closer to the ceiling using environmental data captured at the height of 0.6 m. Time is of the essence in firefighting because firefighters’ PPE and the structural building members deteriorate with time. The longer the firemen or firewomen stay at the fire scene, the higher their chances of getting injured or killed.

The environmental and positional data needed to train the machine learning model are collected from the NIST-CLT (Cross-Laminated Timber) database. These data were modeled to create different scenarios in the burning structure to train the DL models. The output of these models is used to guide how the AESV navigates the fire scene. A High-Performance Engineering (HiPE) research group member designed the AESV to enable wireless communication. It captures data as it navigates the scene and transmits it to a base station in a fire bus outside the burning site for analysis [6]. Since the AESV works independently, all the available firefighters on the fire site focus on firefighting.

This research mainly aims to develop a model to classify fire sites into dangerous levels and predict temperature changes in real-time using deep learning and predictive modeling. This research provides real-time information about temperature and danger levels on the ground, which can reduce uncertainty at the scene and increase firefighter safety. The study uses an open-source dataset from the Fire Calorimetry database, which captures temperature, carbon monoxide (CO), and smoke levels during different fire conditions. The proposed models, including the random forest regressor (RFR) and autoregressive integrated moving average (ARIMA) models, are evaluated and compared to traditional machine learning models. This research can benefit firefighters and other high-risk occupations such as mining, oil exploration, and construction, and future research can focus on improving the performance of the proposed models.

### 1.1. Classification Literature Survey

The authors in [8] proposed using an improved stacked autoencoder (SAE) model based on a deep learning network to refine traffic flow prediction performed by the Intelligent Transport System (ITS). This model improved the traffic flow forecasting accuracy to easily control the traffic with high-dynamic change and various noises in the real world. The SAE model successfully extracts the attributes among the traffic flow data, and the forecasted result shows that the proposed model has better accuracy than other traffic flow prediction methods. This was achieved by using the dropout method to tackle the over-fitting problem faced by other ML models (LSTM, DNN, SVM, and DBN). A greedy layer-wise unsupervised learning algorithm was used to train and enhance the parameters.

The authors in [9] proposed using a stacked denoising autoencoder, SDA, and its extended version for the short-term forecasting of electricity prices. The researchers investigated two types of forecasting, online hourly forecasting and day-ahead hourly forecasting. The results showed that the SDA could predict electricity prices, and the extended version SDA model increases the prediction performance. The extended version combines the concept of Random Sample Consensus (RANSAC) and stochastic neighbor embedding (SNE).

### 1.2. Prediction Literature Survey

The authors in [10] investigated three prediction models for forecasting pest bird distribution density. Their experimental results showed that the random forest regression (RFR) model produced the best performance metrics (MSE = 5.52% and MAE = 1.49%) for predicting the pest bird distribution density of transmission lines compared to linear regression and decision tree regression models. The authors in [11] reported that using the RFR model for near-surface air temperature prediction in the glacier region produced lower error metrics than the actual values. The authors in [12] performed a comparative analysis to predict electrical strength and boiling temperature using MLR, ANN, and RFR models. They reported that RFR outperforms ANN and MLR architectures with better forecasting accuracy and stability. The authors in [13] compared RFR and a backpropagation neural network for a State-of-Change (SOC) estimator, concluding that RFR has higher accuracy in estimating the battery SOC.

The authors in [14] compared the weather forecast results using the autoregressive integrated moving average (ARIMA) and the exponential smoothing method (ETS). They recorded that ARIMA outperformed ETS with an MSE of 0.34%. The authors in [15] used ARIMA to simulate the rule of weather change to forecast time series temperature and rainfall in the next ten years for Shandong province. They concluded that the prediction effect of ARIMA is good, with relatively small errors between the predicted data and the actual dataset. The authors in [16] used ARIMA to develop suitable models to forecast monthly precipitation. They tested the final model and concluded that, based on its high accuracy, it could be used to predict the monthly variations in precipitation and mean temperature of Shiraz. The authors used the ARIMA algorithm in [17] to predict energy consumption for industrial drying systems. They reported that the model predicted temperature correctly with 99.09%, humidity with 98.24%, and energy consumption with 96.31% accuracy. The authors in [18] conducted a comparative study of ARIMA and adaptive neuro fussy inference system (ANFIS) models for predicting weather conditions in Dhaka, Bangladesh. They compared both models using their errors and concluded that the ARIMA model outperformed ANFIS.

The scope of this research is to develop a solution to classify environmental factors into dangerous levels and predict temperature values at heights close to the ceiling of a residential building. We will use a DL model to classify the ecological data into hazardous levels. RFR will generate data for the 2.6 m height beyond the AESV capabilities to capture data. The generated data will train the ARIMA model to forecast temperature values with time.

## 2. Materials and Methods

The fire site is volatile and unpredictable. The volatility and unpredictability makes it a dangerous place to work as there could be falling objects, the structure/building could collapse, and environmental factors, such as temperature, CO, and smoke level, can change within a twinkling of an eye. Knowing when the difference in these factors exceeds the firefighter’s equipment threshold is good, but predicting when this harmful environmental condition is reached is better. Furnishing the chief firefighter with accurate forecasted data would enable better planning and informed decision-making, allowing the firefighters to quickly carry out their tasks while reducing their exposure to risk in the burning structure. For this research, the methodology will concentrate on classifying and forecasting the environmental data.

**Figure 2:**
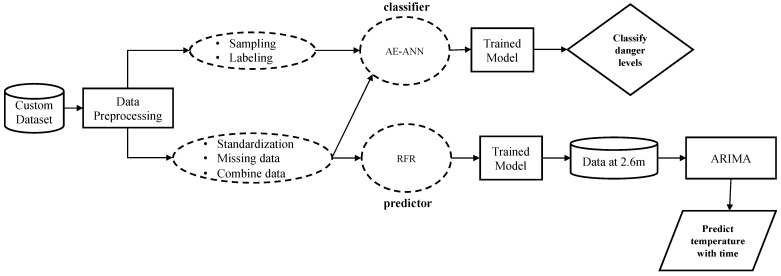
High-level block diagram for predicting firefighter’s safety.

This section discusses the development of the DL architecture for danger level classification of the burning site and prediction models for predicting temperature at heights beyond which the autonomous vehicle can capture temperature data. The DL will dictate the action of the AESV based on its classification of the scene. Depending on the output of the DL models, the AESV will be instructed to either keep navigating the site, slow down, or stop as the situation progresses. This is performed to prevent the AESV from being burnt and to alert firefighters of dangerous spots in the building. Figure 2 shows the overall execution flow of the research design approach.

Environmental factors such as temperature and CO are considered for classifying danger levels and predicting height in a burning structure. Due to the randomness, unpredictability, and dangers of a burning site, performing experiments in these conditions to generate or capture data is almost impossible. The data generated from firefighters’ training sessions would not be helpful for this research because the maximum temperature observed is low compared to what is experienced in an actual fire site. Due to the unavailability of a dataset captured in a real fire scene, we decided to use and customize the dataset from the Fire Calorimetry database [19]. The datasets were recorded during the CLT (Cross-Laminated Timber) compartment fire tests jointly conducted by the National Research Council Canada and the National Institute of Standards and Technology (NIST). The conditions under which these datasets were obtained are like what firefighters experience when they go to rescue victims (people and pets) in a burning site.

The dataset captured during the CLT fire tests consists of temperature levels that show all the fire growth stages, the changes in CO, and the smoke levels as the fire grows and diminishes with time. The appearance and location of smoke give insight associated with the fire’s location and the phase of fire in different compartment locations. Since the conditions in a burning site differ with height and location, and the fire propagates from the source to other cooler areas, the temperature data contain readings taken at different heights and distances of the compartment. Other factors, such as ventilation, that affect the burning site’s conditions were considered in the experimental setup. Ventilation and the nature of the fuel involved affect the fire’s speed and the burning duration [20]. Less ventilation equals lower heat release. For this research, we considered the dataset captured from CLT-Test 1-1 to CLT-Test 1-6.

CLT-Test 1-1 and CLT-Test 1-2 are the baselines with fully protected CLT compartments, while CLT-Test 1-3 to CLT-Test 1-6 contain partially exposed CLT structures. Varying the size of the ventilation and other openings resulted in different times when flashovers and decay occurred. For example, in CLT-Test 1-6, the flashover happened 5 min earlier than in the base experiment (CLT-Test 1-1); though they have similar peak compartment temperatures, the compartment stayed at the peak temperatures until the end, and there was no decay phase during Test 1-6 [20]. Additionally, in CLT-Test 1-2, the larger ventilation opening increased the rate of combustion of the fuels in the room while reducing the intense burning time. This causes the fire to decay earlier in CLT-Test 1-2 than in CLT-Test 1-1. The variability in the dataset obtained from each experiment depicts the different scenarios firefighters may encounter on the fire ground. Table 1 summarizes the purpose of each test and the total number of samples obtained.

**Figure 3:**
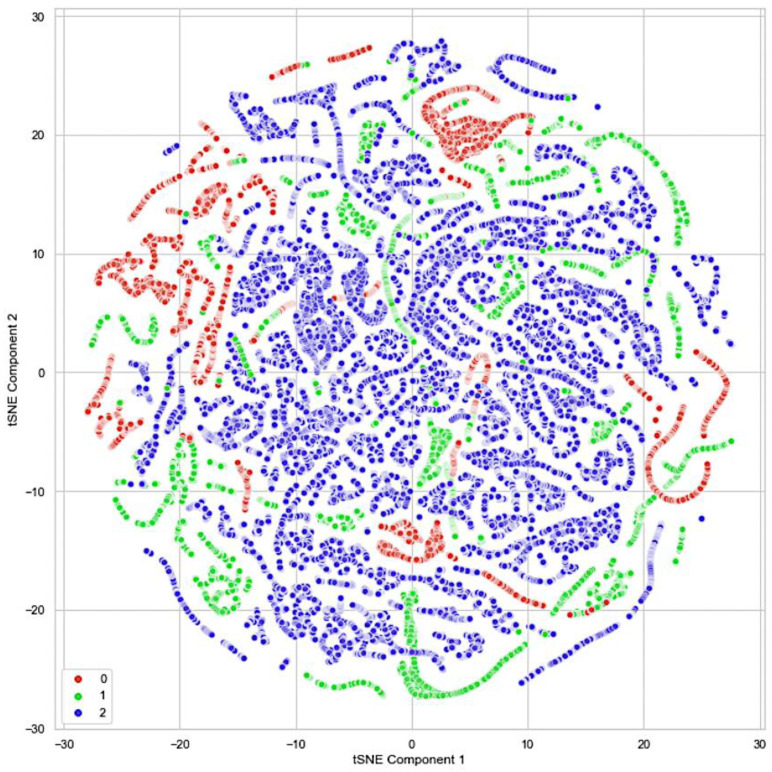
The *t-SNE* plot of the custom dataset of the three different danger-level classes [21].

Figure 3 depicts the randomness and instability of the burning site presented in [21]. The plot shows three different clusters representing the three classes of our dataset. The classes are classified as ‘*safe*’ as zero, ‘*caution*’ as one, and ‘*danger*’ as two. The plot showed some overlapping samples between the three classes. This explains how quickly the fire stages change in a burning site. The t-SNE plot brings to our attention that our dataset has overlapping and imbalance problems, which would increase the difficulty of our trained model in distinguishing between the classes present in the overlapping region and lead to the deterioration of the model’s performance [22].

### 2.1. Custom Dataset

**Figure 4:**
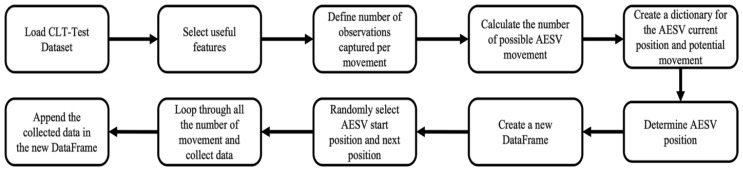
Steps for extracting the custom dataset [21].

The CLT-Test dataset contains thirty different temperature readings captured by sensors at six locations and five heights. Training the DL models with all these readings will result in overfitting. A DL model is as good as the data you train it with. Training the model with 30 columns of temperature readings will increase the computational cost of model training and impair the model’s performance. Since the AESV can only be in one place at a given time and has features that can only capture temperature readings at the height of 0.6 m, we must train the model with sensor readings captured at this height. To mimic how the AESV could navigate and capture data from the actual fire scene, we developed a program in Python to randomly select temperature readings from the dataset from different locations at heights of 0.6 m and 2.6 m. Figure 4 shows the steps for extracting the custom dataset from the CLT-Test dataset and executing the program, which generated two columns of temperature readings containing data from six locations at heights of 0.6 m and 2.6 m.

After loading the dataset, fourteen columns were selected, partially comprising one column of smoke, one column of CO, and six other columns, each representing temperature readings at different parts of the burning compartment at heights of 0.6 m and 2.6 m. We assumed the AESV would capture five observations per movement. The number of possible AESV movements is the total number of observations (data length) divided by the number of observations selected per movement. Next, we created a dictionary of the AESV’s current location and where it could move to, considering the layout of the compartment. A new data frame is designed to store the captured data every time the AESV changes position. After randomly selecting the AESV starting point, the first set of five observations was picked. For every iteration (representing AESV movement), five observations were chosen from one of the six positions of temperature readings at heights 0.6 m and 2.6 m and appended in two different columns, titled *t°C* (0.6 m) and *t°C* (2.6 m), without picking from the same set of observations twice. The iteration continues until the maximum possible AESV movement is attained. We can generate unlimited datasets for each CLT-Test data to train and test the classifier and predictors by running the algorithm as often as we want. Ten other datasets will be generated from each experiment for this research work.

The temperature readings captured by all the temperature sensors at 0.6 m were used to train the DL models to classify the fire scene into dangerous levels because of the height of the AESV. However, to understand the fire progression with height, the predictive models would use temperature readings at 0.6 m and 2.6 m for predicting the temperature as height increases. This enables the AESV to capture readings at 0.6 m and indicates the temperature at the ceiling without having to fly the unit.

The algorithm generates two columns of temperature readings containing data from six areas at heights of 0.6 m and 2.6 m. We used the algorithm to generate ten other experimental datasets for each CLT-Test data. The average of these datasets was taken to create the customized dataset for training the models. This ensures that the random selections capture the population of the dataset. Six customized datasets were created to represent the actual six CLT-Test datasets. In this research, because compartment fires are often different due to factors such as the size of the structure, fuels, ventilation and other openings, availability of sprinklers, and other factors, we used the six experimental datasets (a total of 74,470 samples) to train and test our models, developing a solution that is flexible to different fire conditions.

### 2.2. Data Preprocessing

Data are a fundamental part of DL models. The data quality and the knowledge derived from a dataset impact the learning capacity of any model. Real-world data often contain noises, missing values, outliers, imbalance classes, and non-categorical data, which cannot be used directly for ML models. Hence, it is paramount to preprocess the data before feeding it to the model. Data preprocessing is a data mining approach used to prepare the raw data in a valuable and efficient format to provide concise data for an ML model [23]. Data preprocessing is the key to developing high-quality models. The aim of preprocessing the environmental dataset before they are fed to the classifier or predictor models, either for training or testing, is to improve the data quality in a way that enhances the performance of the models. As shown in Figure 2, the following data preprocessing steps were used to clean and prepare the dataset.

#### 2.2.1. Sampling

Sampling is a technique used to address the problems associated with imbalanced classes. A recurring problem in ML is a disproportionate ratio among class distribution, known as an imbalanced class [24]. Simply put, an imbalanced dataset means the number of observations is not the same for all the classes in a classification dataset. Using an imbalanced dataset to train a model could create a model biased towards the majority class only. The ML models’ performance metrics are optimal when an equal number of samples are present for each category [25]. In this research, we used the random oversampling method on the training data to deal with the problems associated with the imbalanced dataset. This technique generates new synthetic data by randomly duplicating samples in the minority classes. However, random undersampling can also address the class imbalance by randomly removing instances from the majority class. We did not explore this approach in this study, but it could be interesting to investigate its influence on the classifier’s performance in future studies.

#### 2.2.2. Labeling

The dataset was classified into three danger levels based on the firefighter’s equipment threshold, protecting the firefighter against elevated temperature and CO levels. High levels of CO can be expected in every firefighting environment, ranging from 50 ppm to several thousand ppm, depending on the type of fire. Safety is critical for all firefighters responding to a fire call. As firefighters can be at the burning site to rescue victims and extinguish the fire for long periods of time, the firefighters’ safety should be a high priority. According to safety conditions stated by the Occupational Safety and Health Administration (OSHA), the permissible exposure of any human to CO is 50 ppm, which might cause mild neurological impairment after several hours of exposure; the short-term exposure limit of CO is 400 ppm; and the level of CO that is immediately dangerous to life and health is 1200 ppm (fatal after some minutes of exposure) [26,27].

Present-day protective clothing for firefighters can handle temperatures below 300 °C and starts to char at temperatures above 300 °C [26,27,28]. Additionally, the authors of [27] state that the maximum exterior lens temperature a self-contained breathing apparatus (SCBA) can withstand before it degrades is 300 °C. In a burning compartment, the rate of temperature increases could be random and unpredictable. The temperature ranges between 10 °C (at ignition on a cold day) and 1200 °C +. The authors in [28] state no possibility of survival in the vicinity at 500 °C and above. Based on these criteria and the level of protection the SCBA and firefighter’s garment can provide, the dataset was labeled into three danger levels as follows: the CO range from 0 to 800 ppm and temperatures from 0 to 270 °C are labeled ‘*safe*’; 801–1600 ppm and 271–599 °C are labeled ‘*caution*’; above 1600 ppm and 600 °C are labeled ‘*danger*’.

#### 2.2.3. Standardization

The standardization technique was used to scale the range of features of an input dataset to have zero mean and unit variance. It prevents elements with more significant variance from dominating the objective function and hinders the classifier’s ability to learn from other features. Standardization gives all input elements the same influence on the classifier and speeds up the algorithm’s calculation.

#### 2.2.4. Missing Values

The dataset contains a few missing values. Missing data are dealt with by omitting the entire row containing the missing value or replacing it with mean, median, or most frequent observation. Missing values are treated as *NaN*, known as not-a-number in ML. To tackle this, omitting the rows containing the missing values was not an option because it could distort the hidden information the predictor models have to learn in the time series dataset. We used the *SimpleImputer* library from *scikit-learn* to impute the mean value to replace the missing values.

#### 2.2.5. Combing Dataset

Six sets of experimental data were obtained from the CLT-Test. To develop a robust model that can efficiently classify or predict the temperature with time in every scenario captured in the CLT-Test, we trained the classifiers with five sets of experimental data and used one for testing. We used Tests 1-2 as the test data. This dataset was not introduced in training the classifiers. To combine the training dataset for the predictive model, we took the mean of every time series sample to generate a new dataset containing information on all the training datasets. However, for the classifiers, we combine all the Excel sheets containing the datasets into one because they do not require a time series dataset.

### 2.3. Autoencoder

The Autoencoder (AE) is an unsupervised ML technique often used for feature extraction and dimensionality reduction [29]. An AE is a feed-forward neural network and consists of three parts: the input layer, the hidden layer (encoder), and the output layer (decoder) [30,31]. Each layer contains neurons. The reduction in the number of neurons in each layer starts from the input layer and is followed by the mirroring of the layers at the center, known as the bottleneck, to build the autoencoder’s decoding section. An AE aims to constrict the input into a lower-dimensional code and reconstruct the new representation’s output to obtain the initial picture [8,30,31]. Like the multilayer perceptron, the AE framework consists of NN with one or two hidden layers. Compressing the input vectors into lower dimensions increases the learning efficiency [7]. AE’s input and output layers should contain the same number of neurons [8,9] because the AE aims to initialize the latent layer parameters that will reconstruct the multidimensional input data [31].

AE performs similar functions as the principal component analysis (PCA) but is more flexible than it. Unlike the PCA, which serves only linear transformation, the AE can perform linear and non-linear transformations in encoding. AE network reconstructs data using the encoder and decoder functions. AE aims to learn the essential features present in the data by reducing the reconstruction error between the input and output data [30]. We consider using AE to classify the environmental data into three danger levels because the AE learns the essential features present in the data by reducing the reconstruction error between the input and output data. The primary objective of this technique is to investigate if the hidden representation of the data carries enough information to classify the samples into their respective classes efficiently. Thereby reducing the number of dimensionalities needed and increasing learning efficiency [9]. This will increase the computational speed of the model because we do not have to perform the decoding phase of the AE. This makes it suitable for this research because time is of the essence in a fire ground.

The AE in our proposed model accepts an input vector x that belongs to the three-dimensional space x∈R^3^. The input is then passed through six hidden layers. The initial three hidden layers of the AE contain 8, 4, and 2 neurons (source code link is given in Supplementary Materials). Their function is to compress the input data into two dimensions to form the encoded representation. Because we want to investigate the information in the latent representation of AE, we disconnected the decoder after training the model to achieve good reconstruction. In so doing, we achieved a neural network that encodes the input data into two dimensions. Unlike supervised learning, the model we developed does not look for a specific pattern associated with a target. However, it learns to use the input space in any way that maintains the most characteristic and predominant information of the input data to allow good reconstruction in the decoder. We used the Keras functional API in Python to model the AE. In the encoding layers, the input layer describes the dimensionality of the input vector, which is three. The Dense class constructor accepts the neutrons and the activation function as attributes, and the input to the layer is included. As an activation function, we used neurons 8, 4, and 2 for the three layers in the encoder and ‘*tanh*’. The ‘*tanh*’ function suppresses the input value to a bounded range. This restricts the network weights to a range, preventing exploding gradient problems. To classify the latent representation of the AE, we used an artificial neural network, ANN, as the classifier. Table 2 shows the results of changing the number of layers and neurons in the encoder of the proposed AE-ANN model. Four different encoder structures were tested with from 2 to 64 neurons, and the best accuracy was achieved with an encoder structure of 8, 4, and 2 neurons, which achieved an accuracy of 80.96%. The bottom half of Table 2 shows the results of changing the number of layers and neurons in the ANN of the AE-ANN model while keeping the encoder structure constant. Three different ANN structures were tested, with from 4 to 8 layers and from 4 to 32 neurons per layer. The best accuracy was achieved with an ANN structure of 4, 8, and 4 neurons, which achieved an accuracy of 80.96%

The model’s performance was evaluated by experimenting with different activation functions in the hidden and output layers. The models were trained and tested using various activation functions to determine the impact on performance. The *‘linear’* activation function, commonly used in regression tasks, achieved an accuracy of 80.61%. However, it is not typically used in classification tasks due to its inability to model non-linear relationships. The ‘*tanh’* function achieved a higher accuracy of 80.85% and is commonly used in hidden layers of an ANN due to its ability to model non-linear relationships. The *‘relu’* activation function achieved an accuracy of 67.69%, which was lower than the other functions. Based on these results, the ‘*tanh*’ activation function was the best choice among the tested functions for this specific classification task. The *‘softmax’* function was utilized to obtain the normalized probability values of the classification decisions. The results are further discussed in the Results section.

The ANN takes the encoded data as input and classifies them into three danger levels. We used the Keras sequential modeling approach in Python to develop this classifier. The input layer has two neurons with a *‘tanh’* activation function. Three hidden layers were deployed with 4, 8, and 4 neutrons with the *‘tanh’* activation function. The output layer has three neutrons with a ‘*softmax’* activation function. A dropout of 0.5 was used to prevent overfitting. The effect of increasing the size of the epoch and batch size was examined to evaluate its impact on model performance. Several experiments were conducted with different batch sizes ranging from 8 to 64 and epochs ranging from 10 to 200. The model’s accuracy was recorded for each variation of batch size and epochs. The results showed that the model’s accuracy ranged between 78.13% and 80.95% across different batch sizes and epochs. Increasing the epoch or batch size did not significantly change the model’s performance. This lack of significant impact on performance may be attributed to model convergence, architecture, data size, and complexity. For this research, the model achieved optimal performance with a batch size of 50, epochs of 100, and SGD as optimizer with a learning rate of 0.005 and momentum of 0.9.

### 2.4. Random Forest Regression

The random forest algorithm uses the same concept as the decision tree on a larger scale. A random forest consists of multiple unrelated trees used in making decisions [10]. It is an ensemble learning algorithm that creates the effect of classification and regression more effectively than that of a single decision tree. We utilized random forest regression (RFR) to predict outputs with numerical or continuous values. The RFR utilizes several regression trees, each producing a prediction value, and the mean value of all the prediction values is taken as the final output. RFR is used in this research to predict temperature values at the height of 2.6 m, given the CO, smoke, and temperature values at an altitude of 0.6 m (source code link is given in Supplementary Materials). The chief firefighter needs this interpolated data to decide when to call off the rescue operation. In a burning site, the temperature rises rapidly with height, and these elevated temperatures weaken the building structures with time.

We developed the RFR model by importing the random forest regressor from the *sklearn* package of *Python* containing ensemble learning. The main hyperparameter in RFR is the number of estimators used. The estimators are the decision trees, and the more estimators used, the better the predicted value. Several experiments were conducted to determine the optimized n-estimators parameter, determining the number of trees created in the random forest. Using *n*-estimators = 1000 generated the best model performance metrics. The trained RFR model was used to predict the six CLT-Test datasets. Then, we combined the predicted values of the six experiments by averaging them so that the final data used to train the ARIMA model contains components of the six CLT-Test datasets.

### 2.5. ARIMA

As the name implies, the autoregressive integrated moving average (ARIMA) integrates two models: the autoregression model and the moving average model [15]. Unlike the multiple regression model, which forecasts the target variable using the linear combination of predictors, an autoregressive model predicts the variable of interest using a linear combination of historical values of the variable. In simple terms, autoregression is the regression of the variable versus itself. On the other hand, the moving average model uses past forecast errors in a regression-like model [32]. The predictors in an ARIMA model comprise both the lagged values of the variable of interest and lagged errors.

An ARIMA model uses the autoregressive moving average to fit series with stationary time. Typically, the environmental data are non-stationary time series. To use ARMA on this dataset, it must first undergo differencing transformation to convert it to a stationary time series. Hence, the word integrated. The concept of predicting a future time series value using past time series data is known as ARIMA [17,18]. The ARIMA model assumes that the current value of a variable within a time series has a linear relationship with the historical observations of the time series and random errors. Additionally, the random errors are assumed to be independently and identically distributed with an average of zero and constant variance.

The ARIMA model is used in this research to predict the future values of temperature readings at 2.6 m (source code link is given in Supplementary Materials). The fire reaches its fully developed phase at a height of 2.6 m faster than at 0.6 m. This model gives the chief firefighter informed temperature data of what the temperature would be in time *t_n_*_+1_ in the future from past time series data, *t_n_*_−1_. The model is developed to predict the whole fire development stages using temperature data at 2.6 m. We trained the ARIMA with data from the RFR (generated temperature readings at 2.6 m). Several experiments were performed to optimize the p, d, and q values. The p-parameter is the order of the autoregressive part, d is the degree of first differencing, and q is the order of moving average part. The value of d impacts the prediction intervals. The higher the value of d, the more rapidly the prediction intervals increase in size [32]. The optimized p, d, and q values used are 1, 2, and 1, respectively.

## 3. Results

### 3.1. Autoencoder Classifier Analysis

The classifier was evaluated based on classification accuracy, precision, recall, F1-score, and misclassification. The accuracy gives the percentage of correctly classified samples. It measures how well the model performed in predicting samples from the dataset. In a case like ours with the class-imbalanced dataset, in which there is a significant disparity between the number of classes, accuracy alone does not describe the model’s efficiency. We cannot conclude a model’s performance based on its accuracy value because accuracy depends on the class distribution. It does not differentiate between the numbers of correctly classified samples of the different classes, and the number of misclassified samples for each category cannot be deduced from accuracy. For this research, we must know how well the model classifies each sample into its actual classes. A good model for this research would be efficiently classifying the ‘*danger*’ class because this is the class that poses the greatest danger to the firefighters’ safety.

Performance metrics focused on class-independent quantities are more appropriate in choosing the best model. Precision, recall, and F1-score fall into this category. Precision and recall provide insight into a model’s ability to classify individual classes. Precision calculates the ratio of samples classified as positive, which is true-positive. It tries to answer what proportion of true-positive identifications was correct. It considers how both the positive and negative samples were classified. A high precision value denotes a model that makes many correct positive classifications, while a low precision value is that with many false-positive classifications. Recall, on the other hand, measures the percentage of actual positives identified correctly. It measures the model’s ability to classify true-positive samples. A high recall denotes a model that can correctly classify all the positive samples as accurate. Recall and precision are jointly used because the number of examples incorrectly labeled as positive cannot be deduced from recall.

In contrast, precision is sensitive to the distribution of the classes [33]. Both metrics provide knowledge of the model classification efficiency on individual classes. F1-score is interpreted as the weighted, or harmonic, mean of the precision and recall. The values for accuracy, recall, precision, and F1-score for the AE-ANN model when tested on the held-out (test) samples can be seen in Table 3.

Table 3 summarizes the performance metrics for the AE-ANN architecture trained with an imbalanced and balanced dataset. The training accuracy indicates a good classification convergence during the training phase. The imbalanced AE-ANN is the DL architecture that was trained with the actual data without adding synthetic data points, and it recorded the low-performing metrics, as shown in Table 3. Its recall value of 66% means the model struggles with classifying the classes correctly.

**Figure 5:**
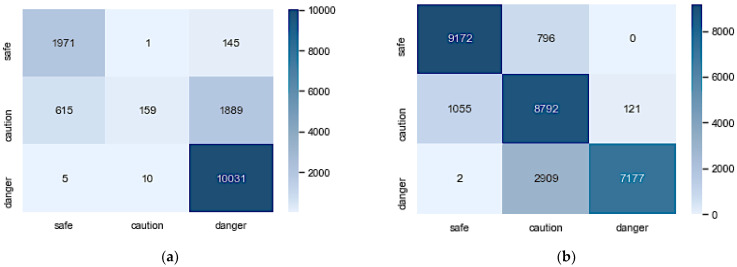
Confusion matrices of AE-ANN architectures trained with imbalanced and balanced datasets: (**a**) imbalanced AE-ANN; (**b**) balanced AE-ANN.

The confusion matrix in Figure 5a,b gives more insight into how the model classifies each class. Figure 5a shows that the model did exceptionally in classifying ‘*safe*’ and ‘*danger*’ samples but struggled with ‘*caution*’. The AE-ANN model trained with an imbalanced dataset could correctly classify only 6% of the ‘*caution*’ samples.

The confusion matrix is a performance metric that separately shows the number of correctly and incorrectly classified examples for each class. It visualizes the performance of the classifiers by showing the numbers of samples of a particular class predicted correctly and incorrectly. Figure 5 shows the confusion matrices for the two models. Figure 5a shows that the imbalanced AE-ANN wrongly classified 1889 ‘*caution*’ and 145 ‘*safe*’ samples as ‘*danger*’. This indicates that the firefighters were falsely alerted to immediately abandon the rescue mission and evacuate the burning site because of their safety 2034 out of 14,826 times (13.72%). This is very high compared to the results reported in [21]. Figure 5b shows an improvement as the number of samples falsely classified as ‘*danger*’ reduced drastically, with 121 ‘*caution*’ samples. However, it increases the number of ‘*danger*’ samples misclassified as ‘*caution*’. This means that 2911 of 10,088 (28%) firefighters will be misled to stay in the burning site when the situation has escalated. The misclassified samples in these models could be because of overlapping or boundary samples. It can be noticed that the two models do not confuse *‘safe’* with *‘danger’* because the boundary conditions for labeling these classes are far apart.

Table 4 summarizes the performance metrics of the classifiers when tested on totally unseen data (EXP2) and other data (EXP3). The EXP2 dataset was not used in developing the training dataset, and it was not used in training the models. It is a new dataset used for testing. The EXP3 dataset components were used to develop the training data. The DL classifier could not classify the data samples correctly, as seen in its performance metrics. This tells us that the latent representation used to train the AE-ANN did not portray the information in the dataset. The poor performance of the DL could be because deep learning models require a considerable amount of data, and we used the latent representation of the data to train the model.

### 3.2. RFR Analysis

Table 5 compares the predictive models in [30] with RFR regarding errors and R^2^. All the models present R^2^ solid values, which represent the proportion of variance in the dependent variable that the predictors have described in the model. The R^2^ values in Table 5 show that the variance in the temperature values at 2.6 m can be explained well by the predictor variable values. This metric must be used with help to determine the best model because it needs to tell us how precise the prediction interval will be.

The mean square error (MSE) measures the prediction error per square in the test data. A low MSE value signifies that the predicted data are close to the observed values. The mean absolute error (MAE) measures the mean of the absolute error values. The root mean square error (RMSE) explains how far off the expected model’s next prediction should be. It gives a good estimator for the standard deviation of the distribution of errors. RFR has the best performance metrics in predicting temperature at 2.6 m. It has better R^2^ values and produces far fewer errors than the MLRs, and, here, we do not have to split the dataset into fire development stages to develop the model. Based on this result, we used the RFR to generate the data to train the ARIMA model.

### 3.3. ARIMA Analysis

**Figure 6:**
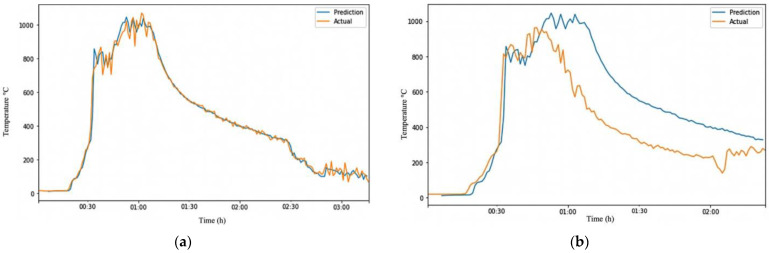
Visualization of ARIMA output on different Test-Data. (**a**) ARIMA output of Test-Data EXP1; (**b**) ARIMA output of Test-Data EXP2.

We fed the RFR-trained model with the data from CLT-Test 1-1. This generated temperature data at the height of 2.6 m. The temperature data at 2.6 m were used to train an ARIMA model. The trained model was evaluated on randomly generated Test-Data EXP1 and Test-Data EXP2 datasets. Testing the model on Test-Data EXP2 is easier because we have the ground truth of CLT-Test 1-2. Table 6 summarizes the performance metrics of the ARIMA model on the test datasets. Figure 6a shows that the model performed exceptionally well in predicting the trend and temperature values of Test-Data EXP1 with a 0.995 correlation, an MAE of 18.79, and an RMSE of 32.78, as shown in Table 6. The results reflect that the ARIMA model was trained with a dataset containing components of CLT-Test 1-1. Evaluating the model on Test-Data EXP2, for which datasets were obtained from a different experiment, proved the ARIMA model’s reliability.

The prediction made by the model was relatively accurate, as shown in Figure 6b, with a correlation of 0.873, an MAE of 167.47, and an RMSE of 206.35. However, the errors would be expected to increase due to the differences between the two fire grounds. The conditions at a fire scene can vary significantly from one location to another and from one time to another, making it invalid to assume that fire behaves consistently. This is especially evident when firefighters move from one room to another. The situation in the new room can be very different from the previous room due to factors such as ventilation and fuel availability.

As seen in Figure 6b, the trained model correctly senses the rising temperature and starts predicting high temperatures. Still, it takes longer for the prediction to match the actual temperature. However, both lines eventually converge and follow the same trend. The issue with this model is that the prediction line (blue) is on the right side of the actual line (orange), indicating that the model is predicting temperature changes (fire development phases) to occur later, which could be dangerous for the firefighters’ safety. It would be safer if the prediction line were on the left side, where the model predicts the fire stages to happen sooner, giving firefighters more time to evacuate the fire ground before conditions escalate. To address the limitations of the above ARIMA model, we combined the six experimental datasets by averaging or taking the median of every data point to create a more robust and flexible model that can better handle variations in fire conditions.

**Figure 7:**
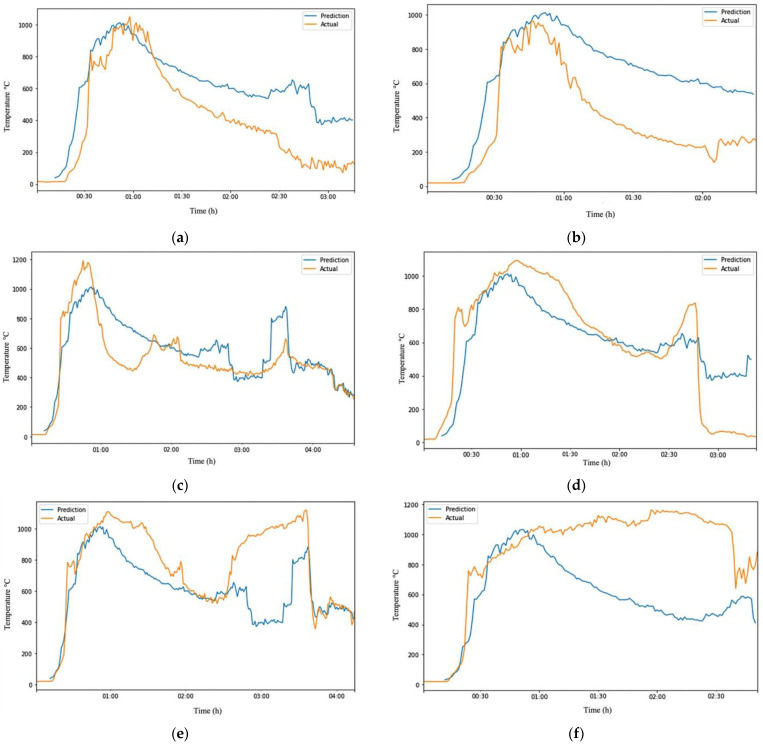
Visualization of robust ARIMA output on different test data. (**a**) robust ARIMA output of Test-Data EXP1; (**b**) robust ARIMA output of Test-Data EXP2; (**c**) robust ARIMA output of Test-Data EXP3; (**d**) robust ARIMA output of Test-Data EXP4; (**e**) robust ARIMA output of Test-Data EXP5; (**f**) robust ARIMA output of Test-Data EXP6.

In Figure 7a–f, the orange line represents the actual dataset, while the blue line represents the predicted data. The ARIMA model trained with one experimental dataset could make relatively accurate predictions on test data with similar conditions, but it is likely to produce significant errors when applied to other experimental test data due to overfitting. The errors in some experiments increased as the model was trained with more observations by taking the average or median of the six experimental datasets to create a more robust model. Overall, developing a model that could perfectly predict fire grounds with different conditions was not possible. An examination of all the outputs of the ARIMA model on datasets that it had not previously seen in Table 7 showed that it was impossible to develop a model that could perfectly predict fire grounds with different conditions. However, it was possible to create a robust and flexible model with a slightly higher error rate that was not prone to overfitting.

The ARIMA prediction model’s results showed that it could accurately predict the trends of temperature changes at a burning site. While there was a discrepancy between the predicted and actual values, the predicted values rose and fell as expected, indicating that the model successfully predicted the trends. At specific points, the prediction line crosses over the actual line, with the prediction being higher or lower than the actual. If the prediction line was above the actual line, the model predicted a higher temperature than the measured one. On the other hand, if it was below the actual line, the model predicted a lower temperature.

In the early stages of a fire, the position of the prediction line relative to the actual line can have different implications. If the prediction line is to the left of the actual line, the model predicts aggressively, suggesting that the fire will reach the fully developed phase sooner. Conversely, if it is to the right of the actual line, the prediction is more conservative, expecting the fire to reach the fully developed phase later. The aggressive predictions made by this model can provide firefighters with the time they need to evacuate the scene safely before the fire escalates and potentially causes an explosion, the building collapses, or fatalities occur. This knowledge can enhance the safety and efficiency of both staff and materials.

However, it is essential to note that aggressive predictions may also result in some victims not being saved if the firefighters choose to evacuate before the fire escalates. In these cases, the risks and benefits must be carefully weighed. Ensuring the firefighters’ safety and health can save them more lives in the future. On the other hand, if the model predicts more conservatively, the firefighters may believe that the site is at a lower temperature than it is, posing an increased risk as they may not be aware of the severity of the fire and the structural damage it has caused. As a result, they may be more inclined to continue working, potentially increasing their safety risk.

We investigated the amount of data that the AESV needs to capture for the ARIMA model to make a good prediction at a fire scene. Time management is crucial in a burning site, and Table 8 shows that as little as 5 min worth of data is sufficient. This means that while the firefighters are putting on their protective equipment (PPE) and gathering the necessary materials for the rescue operation, the AESV can navigate the scene to collect enough data to predict temperature changes at the location.

**Figure 8:**
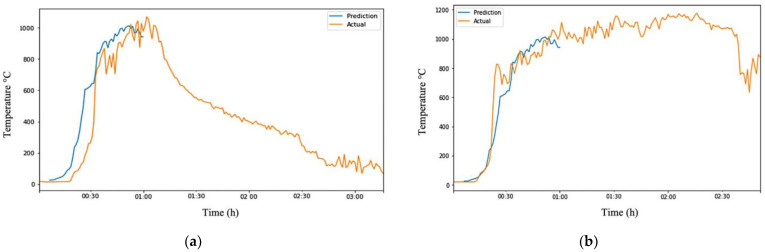
Visualization of robust ARIMA output showing its effectiveness in predicting from inception to the first 60 min. (**a**) Robust ARIMA output of Test-Data EXP1; (**b**) Robust ARIMA output of Test-Data EXP6.

Overall, the ARIMA model showed firm performance in predicting temperature changes from inception to the fire growth stage, as seen in Figure 8. This is likely because most fires behave similarly during this stage, requiring fuel combustion for the fire to propagate. Table 9 summarizes the performance of using 5 min of captured data to predict the next 55 min of temperature changes in different fire ground conditions. On the other hand, the model had more difficulty predicting temperature changes from the fully developed phase to the decay phase as the decay rate depends on various factors, such as the amount of fuel available. Fires with more fuel tend to have a longer decay rate, leading to more significant errors in the prediction.

## 4. Discussion

The dataset used in this research was obtained from the Fire Calorimetry database. The joint effort of the National Research Council Canada and the NIST to investigate the impact of CLT elements on structure fires produced the dataset. They performed six different experiments, which represented six different fire conditions. The conditions under which this dataset was obtained are what firefighters experience when they go to rescue victims (people and pets) in a burning site. The dataset captured during the CLT fire tests consists of temperature levels that show all the fire growth stages, changes in the CO, and smoke levels as the fire grows and diminishes with time. We developed a program in Python to randomly select temperature readings from the dataset from different locations at heights of 0.6 m and 2.6 m. This data selection mimics how the AESV would capture data when navigating the fire ground. The datasets were labeled into three classes based on the firefighter’s equipment threshold, which protects the firefighter against elevated temperatures and CO levels.

A combination of an AE and an ANN was used for the DL model approach. The AE-ANN technique was used to investigate if the latent representation of the data carries enough information to classify the samples into their respective classes efficiently. We have reported an accuracy score of 86.9% in comparison to the three ML models showing a high percentage of correctly classified samples with test accuracies of 92%, 93%, and 99% for its SVM, logical regression, and *k*-NN, respectively, in [30]. The poor performance of the DL could be because deep learning models require a considerable amount of data, and we used the hidden representation of the data to train the model.

The RFR model produced much better results than MLR models for predicting temperature to help us understand the temperature rise with height. The RFR was used to generate temperature data at the height of 2.6 m using environmental data captured at the height of 0.6 m. Thus, eliminating the need to mount an additional ecological sensor capable of capturing data at 2.6 m. The ARIMA model excellently predicts the trend, but the errors are high due to significant variations in the decay phase of fire development stages exhibited by different fire grounds. The results showed that our ARIMA model could predict the entirety of the fire development stages with as little as 5 min worth of data.

This research is vital because it presents a DL and predictive approach to reduce the number of firefighting casualties at the burning sites. In the past, the decision to evacuate the firefighters from burning structures, based on experience and protocol, resulted in some firefighter injuries, entrapments, and fatalities. With these research models’ abilities to efficiently classify burning sites into dangerous levels and predict temperatures, firefighters have informed data of their surroundings. The classification and prediction reduce uncertainty at the scene and increase safety. Firefighting is a daunting profession, and firefighters deserve resources that reduce risks. This technology could benefit firefighters and other high-risk occupations such as mining, oil exploration, and construction.

The next step is to reduce the errors in the ARIMA model. The ARIMA model is limited in its predictions because of the initial conditions of the available dataset. The ARIMA model can be more fine-tuned with more data samples exhibiting different fire conditions. We must use the reward action modeling system known as reinforcement learning to achieve this. Reinforcement learning is an ML technique that teaches an agent via trial and error in an interactive environment using feedback from its actions and experiences. Here, the agent learns from mistakes. With reinforcement learning, the AESV can be taught different scenarios in simulations. The simulations will involve the AESV navigating different floor plans to capture sensor data. The reinforcement learning will teach how to better predict the trends and durations of fire by rewarding the AESV for every correct prediction. If appropriately implemented, this solution can generate more random datasets for the better adjustment of the ARIMA.

## 5. Conclusions

In conclusion, this research aimed to classify burning sites into danger levels using deep learning and to predict the progression of temperature with height using predictive models on environmental factors captured in the burning area. The dataset was obtained from the Fire Calorimetry database and represented the different fire conditions firefighters may encounter when rescuing victims in a burning site. The dataset included temperature readings from different locations at heights of 0.6 m and 2.6 m and CO and smoke levels. An autoencoder-artificial neural network (AE-ANN) was implemented to classify the data into dangerous levels. Still, the results were disappointing compared to those achieved with three traditional machine learning models. A random forest regressor (RFR) model produced much better results for predicting temperature and was used to generate temperature data at 2.6 m. An autoregressive integrated moving average (ARIMA) model was then trained on this generated data and demonstrated excellent prediction of temperature trends. However, the amount of errors was high due to variations in the decay phase of fire development stages exhibited by different fire grounds. Testing the ARIMA model on randomly generated datasets showed that it could predict temperature changes with as little as 5 min of data. Overall, this research demonstrates the potential for using deep learning and predictive modeling to enhance the safety of firefighters and improve their decision-making processes in burning sites. While the AE-ANN model did not perform as well as expected, the RFR and ARIMA models showed promising results. They could be used in future firefighting technologies to provide real-time information about temperature and danger levels on the ground. Further research could focus on improving the performance of the AE-ANN model and developing more robust and flexible models for predicting temperature changes in burning sites with different conditions.

## Figures and Tables

**Table 1:** Purpose of CLT-Test Experiments.

Experiments	Purpose	Samples
Test 1-1	Effect of 1.8 × 2.0 ventilation	11,762
Test 1-2	Effect of 3.6 × 2.0 ventilation	8563
Test 1-3	Effect of 3.6 × 2.0 ventilation and opened wall	16,497
Test 1-4	Effect of 1.8 × 2.0 ventilation and opened ceiling	12,181
Test 1-5	Effect of 1.8 × 2.0 ventilation and opened wall	15,229
Test 1-6	Effect of 1.8 × 2.0 ventilation and opened ceiling and wall	10,238

**Table 2:** Adding Layers and Changing Number of Neurons for the Encoder and ANN.

Changing Encoder (Neurons)	ANN (Neurons)	Accuracy
4, 2	4, 8, 4	67.79%
8, 4, 2	4, 8, 4	80.96%
16, 8, 4, 2	4, 8, 4	67.27%
32, 16, 8, 4, 2	4, 8, 4	67.77
64, 32, 16, 8, 4, 2	4, 8, 4	76.41
Encoder (Neurons)	Changing ANN (Neurons)	Accuracy
8, 4, 2	4	80.57%
8, 4, 2	4, 8, 4	80.96%
8, 4, 2	4, 8, 16, 8, 4	80.62%
8, 4, 2	4, 8, 16, 32, 16, 8, 4	67.33%

**Table 3:** Comparing performance metrics of AE-ANN model trained with imbalanced and balanced datasets on the held-out dataset.

Metrics	Imbalanced	Balanced
Training set:test set	77.5%:22.5%	75%:25%
Data samples in test dataset	14,826 of 65,890	30,024 of 120,096
Training accuracy	82.00%	84.00%
Test accuracy	82%	84%
Misclassified samples	2665 of 14,826	4883 of 30,024
Recall	66%	84%
Precision	84%	86%
F1-score	62%	84%

**Table 4:** Comparing performance metrics of AE-ANN model trained with imbalanced and balanced datasets on new datasets.

Metrics	Imbalanced AE-ANN		Balanced AE-ANN	
	EXP2	EXP3	EXP2	EXP3
Accuracy	82%	82%	84%	84%
Misclassified samples	5845	5821	5845	5821
Recall	33%	33%	20%	33%
Precision	20%	20%	33%	20%
F1-score	25%	25%	25%	25%

**Table 5:** Comparing performance metrics of RFR and multiple linear regression models.

Predictors	R-Squared	MAE	MSE	RMSE
MLR-Up	0.96	63.29	7469	86.43
MLR-Down	0.99	16.06	424.9	20.61
MLR	0.96	40.25	3408.36	58.38
RFR	0.99	5.3	25.89	5.09

**Table 6:** Comparing performance metrics of two test data.

Dataset	Correlation	MAE	MAPE	RMSE
Test-Data EXP1	0.995	18.79	0.069	32.78
Test-Data EXP2	0.873	167.47	0.519	206.35

**Table 7:** Summary of Performance Metrics of Robust ARIMA Model on Six Test Datasets.

Datasets	Correlation	MAE	MAPE	RMSE
Test-Data EXP1	0.9	199.85	1.13	234.32
Test-Data EXP2	0.86	268.38	1.03	299.48
Test-Data EXP3	0.8	106.91	0.228	144.5
Test-Data EXP4	0.81	152.82	1.19	204.29
Test-Data EXP5	0.67	177.1	0.21	256.82
Test-Data EXP6	0.6	295.77	0.3	358.27

**Table 8:** Summary of Performance Metrics of Robust ARIMA Model using 5 Min Worth of Data.

Datasets	Correlation	MAE	MAPE	RMSE
Test-Data EXP1	0.894	195.22	1.1	229.64
Test-Data EXP2	0.854	259.88	1.02	293.33
Test-Data EXP3	0.823	106.65	0.252	144.45
Test-Data EXP4	0.819	152.7	1.23	204.42
Test-Data EXP5	0.72	180.98	0.22	256.55
Test-Data EXP6	0.65	287.55	0.27	345.6

**Table 9:** Summary of Performance Metrics of Robust ARIMA Model in Predicting from Inception to the first 60 min.

Datasets	Correlation	MAE	MAPE	RMSE
Test-Data EXP1	0.953	119.94	0.35	163.91
Test-Data EXP2	0.945	125.51	0.703	169.38
Test-Data EXP3	0.95	120.85	0.421	147.05
Test-Data EXP4	0.91	134.43	0.26	197.78
Test-Data EXP5	0.97	72.55	0.22	95.61
Test-Data EXP6	0.95	73.3	0.19	114.55

## Data Availability

The data source used for this study was obtained from https://www.nist.gov/el/fire-research-division-73300/firegov-fire-service/fire-dynamics (accessed on 5 February 2023).

## Supplementary Materials

The following supporting information can be downloaded at https://github.com/Saishola/Danger-Levels (accessed on 23 January 2023).
